# Evaluation of Provider Screening Practices for Fracture Risk Assessment among Patients with HIV Disease

**DOI:** 10.1155/2021/6672672

**Published:** 2021-04-20

**Authors:** Faryal Mirza, Sabina Zawadzka, Anne Abbate, Michael Thompson, Dorothy Wakefield, Lisa M. Chirch

**Affiliations:** ^1^Division of Endocrinology, Department of Medicine, The University of Connecticut School of Medicine, Farmington, CT, USA; ^2^Division of Infectious Diseases, Department of Medicine, The University of Connecticut School of Medicine, Farmington, CT, USA; ^3^University of Connecticut Health Center, Center for Public Health and Policy, Farmington, CT, USA

## Abstract

People living with HIV are known to have greater risk of low bone mineral density than HIV-negative peers. The reasons for this disparity are multifactorial. To address this increased risk, the Infectious Diseases Society of America (IDSA) released fracture risk screening recommendations in 2015, which differ significantly from recommendations that apply to the general population. A study was conducted at the University of Connecticut to assess for provider awareness and adherence to these recommendations. Electronic surveys were sent to providers, and patients were also surveyed for risk factors and prevalence of low bone mineral density. The results of the provider survey showed low rates of awareness of the IDSA screening recommendations. A substantial proportion of patients surveyed met criteria for low BMD screening but did not have dual-energy X-ray absorptiometry (DXA) ordered by their provider. As an intervention, providers were sent information via e-mail regarding current screening recommendations, as well as notifications if their patient met criteria for DXA screening. A twelve-month follow-up survey showed increased provider knowledge of screening recommendations and improved screening practices. Additionally, the results of a logistic regression analysis of patient factors showed that increasing age and male sex were positively associated with fragility fracture risk. Increased duration of antiretroviral therapy use was associated with a lower likelihood of fragility fracture.

## 1. Introduction

The risk of low bone mineral density (BMD) and its associated complications including fracture are significantly higher in people living with HIV (PLWH) compared to the uninfected population [1, 2]. The mechanisms leading to this disparity are multifactorial; some are likely related to viral factors, others to antiretroviral therapy (ART) [3, 4]. Secondary osteoporosis risk factors (i.e., malnutrition, coinfection with hepatitis C, substance abuse including tobacco, and alcohol dependence) also have a higher prevalence in the HIV positive population [5, 6]. HIV-infected men frequently develop low serum testosterone levels with aging, further predisposing to bone loss [2, 7]. Furthermore, several studies have found that more than 90 percent of PLWH have 25-hydroxy vitamin D deficiency [8].

To address the increased risk of low BMD in HIV patients, the Infectious Diseases Society of America (IDSA) released fracture risk screening recommendations in 2015 [1]. These significantly differ from screening practices recommended for the general population [9] but are consistent with more recent recommendations from the European AIDS Clinical Society [10]. Specifically, all HIV patients older than 40 years old should be screened for fracture risk using the Fracture Risk Assessment Tool (FRAX) [1]. Dual-energy X-ray absorptiometry (DXA) should also be used in addition to FRAX scores in men aged ≥50 years, postmenopausal women, patients with a history of fragility fracture, patients receiving chronic glucocorticoid treatment, and patients at high risk of falls [1]. Additionally, current guidelines suggest inclusion of HIV infection as a cause of secondary osteoporosis in the FRAX questionnaire [10].

Provider adherence rates to the IDSA screening recommendations are unknown, but there are likely many PLWH who meet criteria for screening and are missed. Primary care providers (PCPs) are less likely to be aware of HIV specific guidelines and may trust instead that this will be addressed by their patient's Infectious Disease (ID) provider. Simultaneously, ID providers might assume that, as with the general population, screening would be performed by the PCP. ID providers may also be less comfortable testing and treating any underlying bone disease that they may uncover. Bone health screening in patients with HIV, therefore, represents an area of care in danger of falling between the cracks of primary and specialty care for PLWH.

The goal of this study was to evaluate provider knowledge and adherence to recent recommendations from international societies and to evaluate the efficacy of an intervention designed to improve provider knowledge and adherence at an academic medical center. In addition, we evaluated our cohort of patients to independently assess the prevalence of risk factors associated with fragility fracture in PLWH.

## 2. Methods

An electronic survey was sent to PCPs and ID specialists at the University of Connecticut Health Center (UCHC) to assess provider knowledge of bone health screening recommendations specific to PLWH. After assessing baseline knowledge, an intervention was designed to attempt to improve provider knowledge and adherence to IDSA screening guidelines.

A random sampling of HIV positive patients followed by both PCPs and ID physicians at UCHC who had appointments during the study period gave consent to be included and interviewed. All enrolled patients were screened in accordance with IDSA recommendations. They were also interviewed about their fragility fracture history, medication history, and other secondary risk factors. Electronic medical records were reviewed to determine if prior FRAX or DXA screenings had been ordered or performed, to collect relevant laboratory data, including CD4 counts and quantitative HIV RNA (viral load), and information on exposure to specific antiretroviral medications. Prior FRAX screenings or DXA scans served as markers for baseline provider adherence to IDSA recommendations. If patients met criteria for a DXA scan [1] but one had not been ordered previously, both the patient's PCP and ID providers were notified via e-mail. This e-mail also included information regarding the current recommendations for DXA screening in PLWH.

After twelve months, a postintervention survey was sent out to the same providers who participated in the preintervention survey to evaluate if the intervention had improved provider knowledge. Medical records of the patient participants were revisited to evaluate if adherence had improved in the form of increased numbers of ordered DXA scans.

In addition to the provider intervention described above, data obtained from patient interviews were used to examine the relationship between adult fragility fractures and other covariate risk factors in this population.

Demographics among those with and without fragility fractures were compared using *t*-tests or Wilcoxon rank sum tests for continuous characteristics and chi-square analyses or Fisher's exact test for categorical characteristics. A logistic regression model examined the relationship between adult fragility fracture risk (FFX) (dependent variable) and patient characteristics. Covariates included age, sex, duration of ART therapy, and protease inhibitor exposure.

This study was approved by the UConn Health Institutional Review Board.

## 3. Results

### 3.1. Provider Knowledge

**Figure 1:**
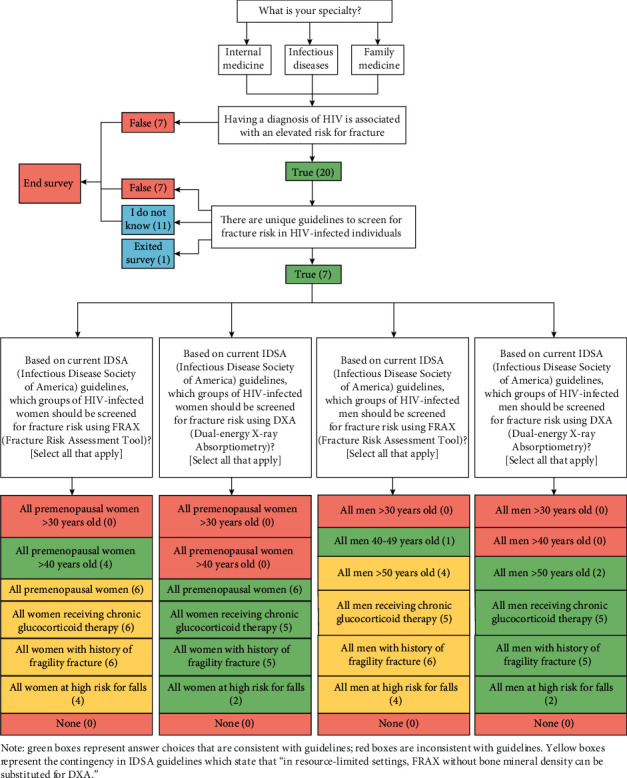
Electronic survey used to assess provider knowledge among PCP and ID specialists. The corresponding (n) refers to the number of providers that selected each choice.

Twenty-seven individuals completed the initial electronic provider survey. Among these, twenty providers (74%; 6/6 ID, 14/20 internal medicine, 0/1 family medicine) were aware of elevated fracture risk in PLWH. However, only seven of those twenty providers (35%) were aware of specific screening recommendations for this population. Additionally, only a small subset of providers was able to correctly identify the current recommendations (see Figure 1). Importantly, 96% of providers were unaware of specific recommendations pertaining to men living with HIV. For the 12-month follow-up survey, response was received from only eighteen providers of the twenty-seven providers who had responded previously. In comparison with the initial survey results, provider awareness of IDSA screening recommendations increased (63% compared to 26%, *p*=0.06, Table 1).

### 3.2. Independent Risk Factors Associated with History of Fragility Fracture

Forty-five patients were enrolled, interviewed, and their medical records reviewed. Baseline characteristics are outlined in Table 2. There were no positive associations identified between immunologic, virologic factors or exposure to specific ART and increased fragility fracture risk (FFR). Overall, 14 (31%) of patients had a history of fragility fractures. This group had significantly higher FRAX scores (*p* < 0.01), and all were recommended for DXA scans. Using logistic regression analysis, increasing age (*p*=0.05) and male sex (*p*=0.06) were positively associated with FFR, trending toward but not reaching statistical significance. Increased duration of ART use showed decreased likelihood of fragility fracture (*p*=0.03, Table 3).

### 3.3. Provider Adherence

Based on the IDSA guidance, thirty-five (35/45, 78%) patients met the criteria for DXA screening, twenty-three (66%) of them being male (see patient demographics in Table 2). Only eight of the thirty-five (23%) PLWH who met criteria for DXA screening had previous DXA testing ordered by their provider. Among the DXAs that had been ordered, a majority were ordered by specialist providers (i.e., endocrinology, infectious diseases). Following the intervention, an additional five patients had DXA screening ordered, which increased the percentage of appropriately screened patients (23% to 37%). Despite this improvement, less than 50% of the eligible patients were screened.

## 4. Discussion

Our results highlight a lack of provider awareness and adherence to the 2015 BMD screening recommendations proposed by the IDSA. All ID providers and more than half of PCPs surveyed were aware of elevated fracture risk in PLWH. Interestingly, a majority within both groups were unaware that specific screening recommendations for the HIV population exist. Even fewer could correctly identify those specific screening recommendations.

The lack of provider awareness regarding BMD screening in our study draws attention to a neglected component of comprehensive primary care in HIV infected individuals. To help improve provider awareness, various modalities can be considered such as educational materials and lectures for providers involved in the care of PLWH, drawing attention to specific factors that place them at higher risk. As highlighted in multiple studies, these include specific ARTs, most notably tenofovir [11–13], along with viral factors leading to chronic immune activation with overproduction of proinflammatory cytokines, eliciting bone resorption [2]. Other nonspecific factors both social and biological likely play important roles, including tobacco and alcohol dependence, low levels of testosterone in males, and of course increasingly overall frailty in an aging population [7].

In this study, our intervention in the form of an e-mail reminder led to enhanced provider awareness and overall patient screening, although a gap remained. It is interesting to note that a larger proportion of HIV positive males compared to females in our study qualified for DXA.

Additionally, male sex was positively associated with FFR, although not reaching statistical significance. Coupled with the finding that almost all surveyed providers were unaware of specific recommendations pertaining to men living with HIV, a concerted effort to increase BMD screening should be promoted surrounding HIV-positive males. Although the association between HIV and increased fracture risk has been widely studied and reported on, there is a relative paucity of data on the extent of adherence to recommendations. Our study highlights an important gap in the delivery of care to PLWH.

The mechanisms behind changes in bone metabolism in PLWH are incompletely understood. HIV infection decreases bone formation and increases bone loss through direct viral effects or indirect effects related to activation of the proinflammatory cytokines resulting in bone resorption and loss [14, 15]. Low CD4 cell counts have been associated with lower BMD, as have coinfection with viral hepatitis [2]. “Immune reconstitution inflammatory syndrome” (IRIS) has also been implicated in the bone loss seen with initiation of ART. The rapid improvement in immune function after the commencement of ART as a result of systemic or local inflammation results in increased levels of cytokines that may contribute to bone loss [16]. Our study did not identify associations with immunologic or virologic factors, or with hepatitis C coinfection, likely related to the small number of subjects evaluated. When assessing risk factors for increased FFR, although some HIV medications have been associated with lower bone density, we did not identify a positive association of lower BMD with ART. Interestingly, increased duration of ART decreased the likelihood of fragility fracture occurrence in our cohort. This differs from prior studies that reported reduced BMD in patients using tenofovir containing regimens [12]. We propose that starting ART is associated with IRIS, resulting in increased bone resorption as mentioned above, but prolonged ART use leading to sustained virologic control may limit bone breakdown linked to viral factors.

This study has several inherent limitations. The low yield of provider response to our survey may not accurately reflect widespread awareness regarding BMD screening. There was a lower response to the postintervention survey, which may incompletely reflect the success of our intervention. Our single center study is also underpowered, and the small sample size may partially explain certain results that differ from prior studies, in particular with regard to secondary osteoporotic risk factors.

There is a critical need for increased provider awareness of BMD screening recommendations and overall BMD screening in PLWH, particularly males. Our small study demonstrates that a one-time intervention to enhance provider awareness improved not only understanding of BMD screening recommendations but also actual BMD screening in HIV positive individuals. It must be emphasized, however, that despite improvements as discussed less than 50% of qualifying individuals were screened, clearly indicating a need for ongoing and innovative interventions. Several factors are likely at play, including persistent lack of knowledge, lack of time, and lack of coverage of BMD screening by insurance companies that may utilize their own criteria. Inclusion of HIV in FRAX calculations as a secondary risk factor may help to further this cause, in addition to ongoing educational efforts at the institutional and public health levels. As the provision of primary care of PLWH expands beyond ID physicians, especially in resource-limited settings, we have an opportunity and an obligation to enhance provider awareness surrounding underutilized screening recommendations that have real potential to tangibly benefit our patients.

## Figures and Tables

**Table 1:** Pre- and postintervention results of faculty survey.

	Preintervention (*n* = 27)	Postintervention (*n* = 18)	*p* value
Elevated fracture risk in HIV infected individuals	20 (74%)	16 (89%)	0.28
Unique guidelines for HIV infected individuals	7 (26%)	10 (63%)	0.06
Premenopausal women >40	4	6	0.17
Postmenopausal women	6	9	0.11
Women receiving chronic glucocorticoid therapy	5	8	0.09
Women with history of fragility fracture	5	9	**<0.05**
Women at high risk for falls	2	7	**0.02**
Men 40–49	1	4	0.14
Men >50 years old	2	8	**<.01**
Men receiving chronic glucocorticoid therapy	5	8	0.09
Men with history of fragility fracture	5	10	**0.02**
Men at high risk for falls	2	7	**0.02**

**Table 2:** Baseline demographics and characteristics.

Variable	Adult fragility fracture (*n* = 14)	No adult fragility fracture (*n* = 31)	*p* value
Age (years), mean (SD)	58.7 (12.1)	51.3 (13.3)	0.08
Male gender, *n* (%)	11 (78.6)	17 (54.8)	0.19
*Race/ethnicity, n (%)*			0.36
** **Black	5 (35.7)	12 (40)	
** **White	8 (57.1)	10 (33.3)	
** **Hispanic	1 (7.1)	7 (23.3)	
** **Other	0	1 (3.3)	
Smoking, *n* (%)	8 (57.1)	20 (64.5)	0.64
ETOH abuse, *n* (%)	3 (23.1)	5 (18.5)	1.0
IV drug use, *n* (%)	2 (14.3)	6 (19.4)	1.0
Diabetes history, *n* (%)	1 (7.1)	4 (12.9)	1.0
Hepatitis C coinfection, *n* (%)	8 (57.1)	10 (32.3)	0.11
CD4 nadir, median (IQR)	472 (375–725)	449 (311–678)	0.45
Virologic suppression >2 years, *n* (%)	11 (78.6)	21 (72.4)	1.0
Years infected, median (IQR)	12 (10–18)	15 (6–26)	0.62
AIDS defined, *n* (%)	4 (28.6)	4 (12.9)	0.23
Months on ART, median (IQR)	39 (29–77)	76 (49–90)	**0.05**
Protease inhibitor exposure, *n* (%)	6 (46.2)%	6 (21.4)	0.11
Tenofovir (DF or AF) exposure, *n* (%)	11 (84.6)	26 (89.7)	0.64
DEXA scan recommended, *n* (%)	14 (100)	21 (67.7)	**0.02**
FRAX score (major osteoporotic fracture), mean (SD)	11.2 (5.8)	5.3 (5.0)	**<0.01**
FRAX score (hip fracture), mean (SD)	1.9 (1.3)	0.6 (0.7)	**<0.01**

**Table 3:** Logistic regression analysis for predicting FFR in patients living with HIV.

Patient characteristic	Odds ratio (95% confidence interval)	*p* value
Age	1.19 (1.0, 1.40)	0.05
Gender (female vs male)	0.10 (0.01, 1.09)	0.06
Months of ART therapy	0.94 (0.89, 0.99)	0.03
Protease inhibitor exposure	12.63 (0.76, 210.04)	0.08

## Data Availability

The data used to support the findings of this study are included within the article.
